# Observer roles that optimise learning in healthcare simulation education: a systematic review

**DOI:** 10.1186/s41077-015-0004-8

**Published:** 2016-01-11

**Authors:** Stephanie O’Regan, Elizabeth Molloy, Leonie Watterson, Debra Nestel

**Affiliations:** 1 Sydney Clinical Skills and Simulation Centre, Royal North Shore Hospital, Level 6 Kolling Building, Reserve Rd, St Leonards, NSW 2065 Australia; 2 Health Professions Education and Educational Research (HealthPEER), Faculty of Medicine, Nursing and Health Sciences, Monash University, Building 13C, Office G09, Clayton Campus, Victoria, 3800 Australia

**Keywords:** Simulation, Observer, Observer role, Directed observer, Vicarious learning

## Abstract

**Background:**

Simulation is widely used in health professional education. The convention that learners are actively involved may limit access to this educational method. The aim of this paper is to review the evidence for learning methods that employ directed observation as an alternative to hands-on participation in scenario-based simulation training. We sought studies that included either direct comparison of the learning outcomes of observers with those of active participants or identified factors important for the engagement of observers in simulation. We systematically searched health and education databases and reviewed journals and bibliographies for studies investigating or referring to observer roles in simulation using mannequins, simulated patients or role play simulations. A quality framework was used to rate the studies.

**Methods:**

We sought studies that included either direct comparison of the learning outcomes of observers with those of active participants or identified factors important for the engagement of observers in simulation. We systematically searched health and education databases and reviewed journals and bibliographies for studies investigating or referring to observer roles in simulation using mannequins, simulated patients or role play simulations. A quality framework was used to rate the studies.

**Results:**

Nine studies met the inclusion criteria. Five studies suggest learning outcomes in observer roles are as good or better than hands-on roles in simulation. Four studies document learner satisfaction in observer roles. Five studies used a tool to guide observers. Eight studies involved observers in the debrief. Learning and satisfaction in observer roles is closely associated with observer tools, learner engagement, role clarity and contribution to the debrief. Learners that valued observer roles described them as affording an overarching view, examination of details from a distance, and meaningful feedback during the debrief. Learners who did not value observer roles described them as passive, or boring when compared to hands-on engagement in the simulation encounter.

**Conclusions:**

Learning outcomes and role satisfaction for observers is improved through learner engagement and the use of observer tools. The value that students attach to observer roles appear contingent on role clarity, use of observer tools, and inclusion of observers’ perspectives in the debrief.

## Background

There has not been a systematic review of the factors that promote learning in the observer roles in simulation. As more learners are allocated to observer roles there is an imperative to ensure that learning in this role is optimised. This review seeks to synthesise the factors that focus the observers’ learning and satisfaction in the role and provide educators with guidance to employing observer roles within their simulations.

Simulation is an effective healthcare teaching strategy [1] and can improve knowledge, skills and behaviours when compared to traditional or no teaching [2]. Simulation conventionally enables learners to physically participate in realistic scenarios replicating real world practice and has been reported as an effective replacement for clinical hours for nursing students [3]. Increasing demand, cohort numbers and access limitations, particularly in professional entry programs has resulted in innovative approaches for learners using simulation. These approaches include role modelling [4, 5], peer and near-peer assisted learning [6–8], and alternative instructional design methods whereby learners are actively directed to observe without hands-on participation [9–11]. We refer to this as the directed observer. When simulation is used appropriately, it improves learning outcomes [2, 12]. However, the evidence supporting learning by observation is less clear.

This review presents evidence supporting directed observation as an educational method and features of this method that lead to positive educational outcomes.

The literature is not always clear on what constitutes observer roles. Here, observer roles are defined as two broad types. First, roles where the learner is external to the simulation. For example, the learner will be watching but not participating in the simulation, either within the simulation area or from an area removed from the simulation. Second, roles where the learner is given a role in the simulation that is not congruent with their professional one. For example, a nursing student could realistically be expected to perform the roles of medication nurse, bedside nurse or documentation nurse in their professional activities. However, they would not be a doctor, social worker or patient relative. In this paper, we describe these roles as ‘in-scenario’ observer roles. Further, observers are described as having a ‘directed observer’ role or a ‘non-directed’ role. A directed observer role would include a specific instructional briefing or use of an observer tool. A non-directed observer watches without specific guidance or objectives. The instructional briefing or observer tool contains information for the directed observer on specific learning objectives, behaviours or activities to consider, points for peer feedback or a checklist to measure against. These specifics would then form part of the debrief.

## Methods

The search was conducted over five databases (Medline, Cinahl, PsycINFO, EmBase and ERIC) within a publication period of 1980 – July 2015 using 45 search terms and restricted to the English language. Hand searching of grey literature, journal contents and reference lists was also undertaken. The study population included any healthcare professional or student who participated in mannequin, simulated patient (actor) or role-play based simulations that included a specific observer role (Table 1). Studies selected included either direct comparison of the learning outcomes of observers with those of active participants following the simulation or identified the factors important for the engagement of observers in simulations and needed to identify their outcome measures and include changes in knowledge, skills, attitudes or behaviours of participants (Table 2) Specific exclusions included computer or virtual reality based simulations as the observer role was difficult to define, and specific task or skill training as the teaching methodology is different than case based scenarios. Video based learning and expert role modelling were also excluded, as there is no comparison of hands-on and observer roles (Table 2).

**Table 1:** Search terms

Population	Intervention	Outcome
Nurs* *or*	Simulation *or*	Learn* *or*
midwif* or	Patient simulation *or*	Knowledge *or*
Medic* *or*	Manikin* *or*	Skill* *or*
doctor or	Mannequin* *or*	Attitude* *or*
surgery or	Simulated patient* *or*	Behav*
Allied health or	Standardised patient* *or*	
Physiotherap* or	Standardized patient* *or*	
Occupational therap* or	Role play *or*	
Dental or	Actor *or*	
Dentist* or	Acting *or*	
Social work* or	theatre	
Respiratory therap* or		
Dietet* or	AND	
Paramedic* or	Observ* *or*	
Aboriginal torres strait	Observ* role *or*	
islander health or	Observational learn* *or*	
Indigen* or	Vicarious learn* *or*	
Inter professional or	Watching	
Interprofessional or		
Intra professional or		
Intraprofessional or		
Multi disciplin* or		
Multidisciplin* or		
Multi profession* or		
Multiprofession*

**Table 2:** Inclusion and exclusion criteria

Inclusion/Exclusion Criteria		
Criterion	Inclusion	Exclusion
Population	Clinicians and students of any health profession	Non health professionals
Intervention	Undergoing a mannequin or simulated patient based learning experience *and*	Computer based, skill or part-task trainers, virtual reality, or cadaveric simulation/simulators.
	• Examines the role of the observer • Has an observer role defined as a learner within a scenario not in a clinically congruent role *or* • Has an observer role external to scenario participant roles	• Studies which do not explicitly examine the observer role. • Observers who are not participating in the learning, for example observers for the purpose of research study. • Expert modelling for learning
Outcome measures	• A direct or indirect change in knowledge, skills, attitudes or behaviours	• Description of behaviours without consideration of any changes in learner behaviour
Citations	Peer reviewed papers in the english language from 1980 to October 4^th^ 2014.	• Non peer reviewed publications e.g thesis or reports • Descriptive papers • Published texts or books

## Results

**Fig. 1:**
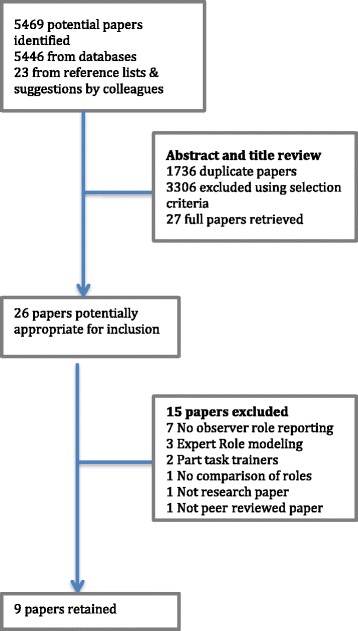
Search flow diagram using the PRISMA process

Nine studies were selected from the 5469 potential papers identified using the PRISMA process [13] (Fig. 1). The studies are summarised in Table 3. The included studies used quantitative, qualitative and mixed methods. A modified version of Buckley’s quality indicators, devised for assessment of quantitative, qualitative and mixed methods studies was selected as the quality assessment tool [14]. These 11 quality indicators relate to the appropriateness of study design, conduct, results analysis and conclusions and are not biased towards any particular research methodology (Table 4).

**Table 3:** Summary of selected studies

Reference	Research paradigm, design & sampling^a^	Participants	Intervention	Learner Observation Style	Results
Bell, Pascucci, Fancy, Coleman, Zurakowski and Meyer [24]	Mixed methods	Health professionals from four disciplines (*n* = 192)	Use of improvisational actors in difficult conversations to teach communication and relational skills to practicing health professional	Non-directed role: no use of observational tool or verbal guidance reported	No difference between observers and hands on learners in: perceived realism; usefulness of actors; usefulness of scenarios; and, opinions on non-actor role play
	Post-simulation survey design with qualitative and quantitative analysis	Teaching faculty (*n* = 33)			
	Convenience sample	Actors (SP) (*n* = 10)			
		Hands on participants (47 %)			
		Observers (53 %)			
Harder, Ross and Paul [25]	Ethnographic stud	Bachelor of Nursing students year 3 (*n* = 84)	Role assignment within regular simulation session with analysis of experience and perceptions of learning within different role	Non-directed role: no use of observer tool or verbal guidance reported	Students preferred assignment to nursing roles rather than observer or non nursing role
	Observational design with focused interview and journal review of selected participants	Participant/observation (*n* = 84) interview (*n* = 12)	All participants experienced both roles		Structured role descriptions positively affected learning outcomes
	Volunteer sample	journal review (*n* = 4)			
Hober and Bonnel [11]	Qualitative	Bachelor of Nursing “senior” students (*n* = 50)	Immersive simulation scenarios with students randomly assigned to active or observer roles	Directed observer role: observer tool – educator provided activity guidelines	Observer role beneficial, less stressful
	Survey and interview design	Observers (*n* = 23)	All completed survey		Use of a guided observer tool useful
	Convenience sample	hands on learners (*n* = 27)	Observers interviewed		Able to reflect in action and on action
Kaplan, Abraham and Gary [27]	Quantitative	Bachelor of Nursing “junior” students (*n* = 92)	Immersive simulation scenarios -	Directed observer role: observer tool -checklist	No difference in knowledge
	Randomised groups	Observers (*n* = 46)	participants self selected roles		
	Convenience sample	Scenario participants (*n* = 46)	Unclear whether observers self selected or were assigned		Limited as aggregated post satisfaction survey data
			Post scenario knowledge test and satisfaction survey		
Lau, Stewart and Fielding [22]	Quasi experimental randomised to roles	Medical students (bilingual) year 1 (*n* = 160)	Student role plays with comparison of learning between interpreter role play and observer role	Directed observer role: observer tool -checklist	Observers rated post knowledge higher than learners in interpreter role-play
	Convenience sample		Self rated pre & post knowledge		
Smith, Klaassen, Zimmerman and Cheng [26]	Mixed methods with increasing variables over three years	Bachelor of Nursing “junior” students	Introduction of simulation year 1	Non-directed role: no use of observational tool or verbal guidance reported	No significant difference in learning outcomes, student perceptions or peer evaluations
			Introduction non nursing participatory roles year		
	Convenience sample	year 1 (*n* = 67)	Introduction non participatory observer roles year 3		
		year 2 (*n* = 72)			
		year 3 (*n* = 85)			
		Note only the year 2 and 3 data were included in review			
Stegmann, Pilz, Siebeck and Fischer [20]	Quantitative	Medical students (*n* = 200)	Comparison of participatory role and observer role in simulated patient scenario with and without observation tool	Non-directed and directed observer roles compared: checklists and feedback scripts used	Observational learning (especially if supported by observer script) more effective than learning by doing
	Crossover design 2x2x2 pre-test post-test				
Stiefel, Bourquin, Layat, Vadot, Bonvin and Berney [23]	Quantitative	Medical students (masters level) (*n* = 124)	Individual training with simulated patient encounter	Non-directed role: no use of observer tool or verbal guidance reported	Measured outcomes no difference
	Randomised into 2 group	Individual training (*n* = 49) Group training (*n* = 75) -participated in simulation (*n* = 14) observed (*n* = 61)	Group training with simulated patient encounter		Those who observed but did not participate felt they did not meet their learning objectives as well compared to the other 2 groups
	Evaluation using instructor rating scale and student questionnaire		Group training with observation of simulated encounter		
	Convenience sample				
Thidemann and Soderhamn [21]	Quasi experimental	Bachelor of Nursing student year 2 (*n* = 144)	Immersive mannequin simulation with random allocation to groups	Directed observer role: observer tool with specific task focus	Post-test scores higher in all groups independent of rol
	Pre - and post-simulation knowledge test and student questionnaire		Four volunteers within each group allocated to participatory and in scenario observer roles – remainder observers (*n* = 72)		More satisfaction with nurse role
	Convenience sample over two consecutive years				

^a^as attributed by author where available

**Table 4:** Study ratings using Buckley's (modified) criteria

Criteria (Yes, No, Unclear)	Bell	Harder	Hober	Kaplan	Lau	Smith	Stegmann	Stiefel	Thidemann
Note: rater disagreement shown as two scores									
Clear research question	U	Y	Y	Y	U	Y	Y	U	Y
Subject group appropriate for study	Y	Y	Y	Y	Y	Y	Y	Y	Y
Reliable and valid methods (qualitative or quantitative) used	Y	Y	Y	Y	Y/U	Y	Y	Y	Y
Completeness of data (drop out, questionnaire response rate >60 %, attrition rate <50 %)	Y	Y	Y	Y	N	N	Y	N	Y
Controlled for confounders or acknowledged if non RCT design	N	U/N	N	U/N	U	N	Y	U	Y
Statistical and other analysis methods appropriate	Y	Y	Y	Y	Y	Y	Y	Y	Y
Data justifies the conclusions drawn	Y	Y	Y	Y	N	Y	Y	U/N	Y
Study could be replicated	Y/U	Y	Y	Y	U	N/U	Y	Y	Y
Prospective study	Y	Y	Y	Y	Y	Y	Y	Y	Y
Relevant ethical issues addressed	U	Y	Y	N	U	Y	Y	U	Y
Triangulation of data	Y	Y	Y	Y	N	Y	Y	N	Y
Total Score/11 (lowest score reported)	7	10	10	9	3	8	11	5	11

Two reviewers (SO, EM) rated the quality of the studies with an inter-rater agreement of 0.94 across 99 data points. Seven studies meeting seven or more criteria as specified by Buckley, were considered high quality studies [14]. There was a wide range of quality with scores from 3 to 11 out of a possible 11. Most common problems encountered were with data completeness, control for confounders, study replicability and addressing ethical issues. Two studies, Stegmann [15] and Thidemann, [16] met all 11 criteria. Two studies, Lau [17] and Stiefel, [18] met six or less criteria. Rater differences are shown in the table as two scores, with the lowest total score reported where there was a discrepancy (Table 4).

To provide composite data the nine included studies were examined using categories adapted from Cook et al [2]. There were a total of 1203 participants across the nine studies with the majority of studies focusing on undergraduate students in nursing (*n* = 527) and medicine (*n* = 484). There was one interprofessional study involving practising clinicians across four disciplines [19]. Five studies used mannequin-based simulations [11, 16, 20–22], two employed simulated patients [15, 18], one an actor [19], and one study involved role-play by the participant group [17] (Table 5).

**Table 5:** Characteristics of included studies

Study Characteristics	Number of Studies	Number of Participants
All studies	9	1203
Study participants		
Medical students	3	484
Nursing students	5	527
Practicing clinicians	1	
Physician		43
Nurse		114
“Psychosocial clinicians”		20
Medical interpreter		14
Study settings		
Mannequin based simulation (high fidelity simulation - HFS)	5	527
Simulated patient (SP)	2	324
Actor (improvisation rather than scripted SP)	1	192
Role play by participant group	1	160
Study design		
Post test only (Knowledge)	1	92
Pre-test/post-test 1 group	1	157
Pre-test/post-test 2 groups	2	344
Self-assessment pre-test and post-test	3	476
Self-assessment post-test only	1	84
Observer role allocation		
Randomised	5	643
Self allocation	1	84
Unclear	2	284
Outcome		
Knowledge	6	869
Skills - technical	3	441
Skills - non technical	8	1059
Attitudes	2	134
Behaviours	1	84
Learning outcomes by role		
Participatory role better than observer	2	208
Observer role better than participatory	1	200
No difference	4	588
Satisfaction by role		
Participatory role more valued than observer	2	208
Observer role more valued than participatory	1	144
No difference in value	3	334
Observational tool used	6	803
Debriefing/feedback		
Observer led pairs	1	200
Faculty led group debrief	7	811
Feedback guide	1	200

Eight of the nine studies compared knowledge, skills, attitudes or behaviours between the hands-on role and the observer role [11, 15, 17, 18, 20–22]. Six studies used a pre and post-test design, three of which were self-assessment of improvement in knowledge and/or skills [17–19] and three studies tested knowledge [15, 16, 21]. Two studies examined knowledge in a post-test only design [22] one of which was a self-assessment [20]. Outcomes included knowledge (six), ‘non-technical skills’ (eight), technical skills (three), attitudes (two) and behaviours (one).

Four studies found no difference in outcomes between the hands-on learners and the observers [11, 16, 19, 22]. Two studies reported superior outcomes in the hands-on group [18, 20] and one study reported better outcomes in the observer group [15]. The study that found superior outcomes for the observer group and three of the four studies that found no difference in outcomes between the hands-on and observer groups [15–17, 22] incorporated an observer tool to guide the observer group. Neither study that demonstrated superior outcomes by the hands-on learners employed an observer tool [18, 20].

Six studies considered the perceived value of the hands-on learner and observer roles to the participants. Two studies reported that participants valued the hands-on roles more than the observer role [18, 20], one study highly valued the observer role [16] and three studies reported no difference in the value of the roles [11, 19, 22]. Two of the three studies with no value difference in roles [11, 22], and the study that valued the observer role highly [16] used an observer tool. The study that valued the hands-on roles higher did not employ an observer tool for the observer group [18]. The observer tools included performance checklists [15, 17, 22], feedback or observation guides [11, 15], or observer role instructional briefing [16]. All studies except Bell [23] documented including observers in the post simulation debrief or feedback.

## Discussion

We sought reported factors that contribute to the optimisation of learning in the observer role. It is clear from this review that the use of observer tools to focus the observer and role clarity are strongly associated with role satisfaction and learning outcomes in observer roles. This finding is supported by Bandura’s social learning theory and Kolb’s experiential learning cycle and we propose that these form the basis of the directed observer role.

One of the outstanding findings from this review is the association of observer tools with both satisfaction and equal if not better, learning outcomes in observer roles. The use of these tools may move observers from simply watching to actively observing. The activation of observers allows those in that role to experience the satisfaction and learning normally associated with hands-on experience. Simulation is described by Dieckmann et al as a social practice where people interact with each other in a goal orientated fashion [24]. The observer tool provides this necessary goal orientation for observer roles. Directed observers are focused on the learning objectives of the simulation.

This is explained by Bandura’s social learning theory, which proposes that virtually all learning acquired experientially could also be acquired on “*a vicarious basis through observation of other people’s behaviour and its consequences for them”* [25]*.* Through observation learners can build behaviours without trial and error, experience emotions by watching others and resolve fears through other’s experience. Bandura describes this as a process of attention, retention, reproduction and motivation [25]. Bethards reports on a program where “*simulation experiences are designed around the observer role using the four component processes of Bandura’s observational learning construct*” [26]. They postulate that this provides all their learners, regardless of role, the same opportunities to achieve the learning objectives [26].

Vicarious learning requires active listening, reflective thinking and situational engagement [27]. Nehls describes this in the context of narratives; lived experiences shared for the purpose of learning [27]. The addition of “active watching” to Nehls’ definition fits well in the simulation context. In a review of vicarious learning, Roberts concludes that vicarious learning occurs during story telling and discourse, and may require a teacher to help find meaning [28]. In the context of scenario-based simulation the story is the scenario or case; active listening and watching is engaged with the use of tools or tasks and the reflective facilitated discussion is the debriefing. It seems important that for optimal learning to occur, observers be engaged in all aspects including the debrief.

Experiential learning is viewed as fundamental to simulation and clinical practice [29, 30] and the theoretical foundations of simulation are commonly described in terms of Kolb’s experiential learning cycle [29]. Kolb proposes a cycle of concrete experiences which on reflection are distilled into abstract concepts that can then provide the basis for future actions and further testing [31]. Kolb stresses that this is an unending cycle and educators need to be aware that learners have a preference for, and may enter at different stages of the experiential learning cycle, but need to be moved through the entire process. A dangerous presumption for educators and learners alike is that concrete experience requires hands-on participation. Vicarious learning theory and Kolb’s experiential learning cycle form the theoretical basis for directed observation.

It seems that observers with the appropriate tools can benefit vicariously from the experience of the hands-on learners. Simulation is a facsimile of the clinical environment so the findings here may also translate to observation in similar clinical practice situations. This directed observer role is different to indirect workplace learning described by Le Clus, where the emphasis is on observers seeking learning to meet their personal needs [32]. However, the concept of observer learning as a social practice aligns with both [24, 32].

Stegmann reports better outcomes from observers preparing to provide feedback than those completing a checklist or in a hands-on role [15]. The impending ‘debrief’ where observers have an expectation that they will be asked to contribute their opinions about the encounter may sharpen the focus of their observations. Bandura describes this as an external motivator [25]. This ‘heightened state’ may mean observers are more likely to engage in standards of practice required for the simulation (for example, measures of good communication) and consider how the simulation participant’s performance measures up to this standard. Thidemann used reporting on standards of practice in her directed observer role guidelines [16].

The learners who did not value observer roles as highly as a hands-on role described observer roles as passive, or boring [20]. They were not fully engaged in the learning process. Emotional engagement in simulation is connected to the feeling of relevance of the scenario to the goals of the session [24]. Lack of goal direction may have prevented observer engagement. It is not clear whether there is an optimal level of activation for learning in observer roles or whether it differs between learners. Learners that valued observer roles described it as being less stressful and providing them the opportunity to see the big picture, examine details from a distance, and provide meaningful feedback to the team [11]. Stress decompression, a feature of debriefing frameworks, is necessary for reflection [30, 33].

The ability to reflect is important in the provision of feedback. An understanding of performance requirements and a judgement regarding the observed performance and its relationship to the standard is required before bridging strategies can be formulated [34]. In directed observer roles, information was provided in the form of the observer tool (e.g. checklist) defining the standards and/or objectives for the learners. The directed observers were able to use these tools to observe, reflect upon and formulate their peer feedback for the debrief.

In-scenario observers, that is non-clinical or other professional roles within the scenario, reported that lack of scripts or clear direction detracted from the act of observation because of anxiety regarding role performance requirements [20]. These aspects of role fidelity have been identified as a barrier to student satisfaction with role play [35]. The other studies that used non-clinical or other non-congruent professional roles viewed these learners as hands-on participants and did not include specific findings for these in-scenario observer roles [17, 20, 21]. Thidemann commented that the nursing roles in their scenarios were the most preferred roles [16]. The lack of clarity in the separation between professionally congruent and incongruent hand-on roles in these studies prevents drawing any real conclusions from the data. In a report of a large study for the National League of Nurses Jefferies and Rizzola 1 concluded that whilst knowledge and self-confidence were unrelated to role allocation, there was a perceived lack of collaboration in the observer role and there was a responsibility for educators to provide structure for this to occur [9].

While learners have assessed the value of observer roles, there has not been a published assessment of the value placed upon observer roles in simulation by educators or facilitators. Use of observer tools or activities and the active involvement of observers in the post-scenario debrief could be considered an indirect indication of the value educators place on learning in observer roles.

It is also unclear as to whether there is a group of learners better suited to learning through observation than learning through hands-on participation in the simulation. Whilst most of the studies used role allocation, one study [20] had a portion of study participants who either self allocated or worked through the case as a group without assigned roles. There was confusion amongst the students in this study as to which roles were considered to be observers; for example some students viewed the documentation nurse as an observer role while others viewed it as a hands-on role. No studies examined whether self-allocation to roles would result in better learning outcomes. The reasons behind self-allocation were also not examined and may be worthy of further study.

An important area for further study includes establishing educator perceived value of observational roles, and the potential impact of these perceptions on simulation education design and orientation of learners to roles within the scenarios. Activation and emotional engagement in the observer role has also not been explored, and provides future research potential.

### Limitations

This review examines one small area of observational learning within scenario-based simulation. Skills training, which is often taught in groups was not included. Also excluded were non peer-reviewed reports, including a major study of more than 400 nurses [9]. This report did however inform the discussion. We also narrowly defined simulation modalities excluding virtual reality simulations where there is even more blurring of boundaries between hands-on participants and observer roles. In some studies it was unclear how the authors defined the in-scenario roles. Reporting of observer roles was in some cases a secondary finding. Lack of clarity may have biased findings. The small number of included papers also limits the conclusions.

## Conclusion

Learning outcomes for participants and observers in simulation can have value if all roles involve active learning either through hands-on roles within the simulation, or through use of tools to facilitate active observer learning. The value that students attach to observer roles seems to be related to the value educators place on them as evidenced through role briefing, use of observer tools to hone judgement of performance compared to standards, and inclusion of observers’ perspectives in debriefing.

## Footnotes

1 This study was not included, as it did not meet the inclusion criteria of peer-reviewed publication however the findings are important and inform the discussion (see study limitations).
